# The Effect of *Thymus vulgaris* on Hepatic Enzymes Activity and Apoptosis-Related Gene Expression in Streptozotocin-Induced Diabetic Rats

**DOI:** 10.1155/2022/2948966

**Published:** 2022-03-23

**Authors:** Mohammadreza Azimi, Jalil Mehrzad, Elnaz Ahmadi, Mohammad Orafei, Fatemeh Aghaie, Armita Ahmadi, Maryam Rahimi, Ali Ghorbani Ranjbary

**Affiliations:** ^1^Department of Biochemistry, Medical Faculty, Saveh Branch, Islamic Azad University, Saveh, Iran; ^2^Department of Microbiology and Immunology, Faculty of Veterinary Medicine, University of Tehran, Tehran, Iran; ^3^Faculty of Chemistry, Islamic Azad University of Tehran, Tehran, Iran; ^4^Research Institute of Agricultural, Ferdowsi University of Mashhad, Mashhad, Iran; ^5^Department of Animal Sciences and Biotechnology, Faculty of Life Sciences and Biotechnology, Shahid Beheshti University, Tehran, Iran; ^6^Faculty of Agriculture, University of Zabol, Zabol, Iran

## Abstract

Many diseases, including diabetes, are involved in the development of liver disorders through changes in the expression of genes such as apoptosis-related genes. In the present study, the effect of *Thymus vulgaris* (*T*. *vulgaris*) on hepatic enzyme activity and apoptosis-related gene expression in streptozotocin (STZ)-induced diabetic rats was examined. In this study, 50 adult male Wistar rats weighing approximately 200–220 g were divided into five groups. Diabetes was induced by an intraperitoneal injection of STZ (60 mg/kg). Following 18 days, all the animals in different groups were weighed and blood samples were taken from their cardiac veins. Gas chromatography-mass spectrometry (GC-MS) analysis revealed 45 different compounds in the *T*. *vulgaris*, including thymol (39.1%), p-cymene (20.63%), and *γ*-terpinene (14.85%). The results showed a significant increase in liver enzymes (aminotransferase (AST), alanine aminotransferase (ALT), and alkaline phosphatase (ALP)) in diabetic or STZ mice compared to the control group (healthy mice) (*P* < 0.0001). The levels of AST, ALT, and ALP in rats treated with 200 mg/kg and 400 mg/kg of *T*. *vulgaris* extract showed a significant decrease in these enzymes in comparison with diabetic rats (*P* < 0.0001). The expression of caspase 3 and 9 genes in the groups treated with thyme significantly decreased compared to diabetic mice (*P* < 0.0001), and the expression of B-cell lymphoma-2 (Bcl-2) in the group receiving 400 mg/kg of thyme significantly increased compared to diabetic mice (*P* < 0.0001). Due to its antioxidant compounds, thyme improves the liver tissue cells in STZ-induced diabetic mice by reducing caspases 3 and 9 as well as increasing Bcl-2.

## 1. Introduction

Diabetes is a common metabolic disorder in humans, which is associated with significant morbidity and mortality, and is a contributor to the development of other diseases. Different genetic factors lead to type-I and type-II diabetes mellitus. Both of them are prone to complications such as nephropathy, retinopathy, peripheral nerve disorders, and blood pressure [1, 2]. Diabetes is the leading cause of hepatic disorders in the USA. Many studies have shown that liver disease is an important cause of morbidity and mortality in type-II diabetes (T2DM) [3]. Besides the high prevalence of liver disease in diabetic patients, the incidence of diabetes is higher in patients with liver disease. It seems that the incidence of liver disease in T2DM occurs due to complications such as abnormal liver enzymes, nonalcoholic fatty liver, cirrhosis, and hepatocellular carcinoma, and acute liver failure is more prevalent [4].

Nonalcoholic fatty liver disease (NAFLD) and T2DM often coexist [5]. The prevalence of NAFLD is 59.67% in T2DM patients [1]. This results in adverse outcomes such as higher rates of mortality due to cirrhosis [1]. NAFLD includes a spectrum of pathological conditions, which range from simple steatosis (NAFL), nonalcoholic steatohepatitis (NASH), cirrhosis, and hepatocellular carcinoma [6].

STZ is a drug used in chemotherapy, and it also specifically kills pancreatic beta cells, thereby lowering insulin levels [5]. The STZ model is a common method for inducing experimental diabetes in rodents and has been used repeatedly in various studies to induce type-I diabetes [6, 7]. Recent studies have shown that STZ causes inflammation by disrupting the relationship between reactive oxygen species (ROS) production and the radical scavenging effect [8, 9]. The rise in the production of free radicals or ROS formation may result in oxidized low-density lipoproteins (LDL), a crucial step in the chain of events leading to atherosclerosis sustained hyperglycemia and increased oxidative stress, which are the key players in the development of the secondary diabetes problems. These abnormalities result in pathologies such as vasculopathies, neuropathies, ophthalmopathies, and nephropathies, among various other medical problems [10]. Through *in vitro* cell culture as well as *in vivo* diabetic rodent models for STZ-induced toxicity, it has been shown that STZ induces cellular oxidative stress and mitochondrial respiratory dysfunction [11–13].

Beta-cell mass decreases in T2DM, and its underlying mechanism is increased beta-cell apoptosis. Because the major defect in T2DM leads to a decrease in beta-cell mass and increased apoptosis. When beta-cell mass formation is appropriate, therapeutic approaches through apoptosis inhibition can be promising for T2DM [14].

STZ induces apoptosis through both caspase-dependent pathway (through the activation of caspase-3 and caspase-9) and caspase-independent pathway (by DNA fragmentation and PARP activation) [15].

A number of experiments have been conducted to assess the changes in cell mitochondrial functions in the brain, heart, liver, and kidney of diabetic rats [14]. Nevertheless, the results have been at times controversial for the reason that experimental conditions such as age and strain of the used animals have been different.

Synthetic drugs for liver diseases, including corticosteroids, antiviral agents, and immunosuppressant agents, might cause serious adverse effects up to hepatic impairment, such as cholestatic jaundice with azathioprine and elevation of serum transaminases by interferon and virazole [15]. As such, it is of paramount importance to explore other sources to treat liver disease more effectively and safely.

*Thymus vulgaris* (called “wild thyme” in Persian) is a plant from the *Labiatae* and *Plantae* families [16]. It grows in many parts of Europe, particularly in southern Europe, north of Africa, as well as large parts of Asia. Wild thyme is used in traditional medicine as an antiseptic, antispasmodic, antiworm, and carminative as well as for relieving liver and bile problems [17–20]. Wild thyme has 1% oil of which a large part consists of phenols, monoterpene hydrocarbons, and alcohol. Wild thyme essence generally contains 25 compounds such as carvacrol (40.7%), thymol (26.9%), and *γ*-terpinene (7.3%) [21, 22]. This research aimed to establish the protection role of *T*. *vulgaris* on hepatic enzyme activity and apoptosis-related gene expression in STZ-induced diabetic rats.

## 2. Materials and Methods

### 2.1. Collecting and Identifying the Plant

Leaves and twigs of the wild thyme plant were collected from the areas near Shiraz, Iran, in March-April 2019, dried at 25°C in the shade, and then powdered by a mechanical mill. The dried powder was kept in plastic bags in a freezer until testing.

### 2.2. Extraction Method

Twenty grams of the obtained powder was poured into an Erlenmeyer flask, and 200 ml of ethyl alcohol at 70°C was added. Next, the flask was closed by its cap and the solution was held for 48 hours, while the content of the flask was shaken once every 12 hours. Following 48 hours, the content of the flask was filtered into a beaker using filter paper and funnel glass. Then, the filtered solution was poured into a flask and placed in a rotary device at 75°C with an average rotation speed. After solvent evaporation, the resultant concentrated liquid was spread on the glass surface and left to dry. The obtained powder containing approximately 2.59 percent of the concentrated extract was collected after drying. Finally, the yielded powder was used to prepare doses of 100, 200, and 400 mg/kg. All the solutions were prepared with distilled water.

### 2.3. Plant Compounds

First, *T*. *vulgaris* essential oil was prepared and the compounds were then isolated by GC/MS device (HP-6840/5973) in the central laboratory of Ferdowsi University of Mashhad. The constituting elements were identified by comparing their mass spectra with the existing standard spectra.

### 2.4. GC-MS Analysis

The analysis of the volatile constituents was run on a Shimadzu QP-2010 GC-MS system equipped with AB-INNOWax 7031428 WCOT column (60 m × 0.25 mm × 0.25 *μ*m) directly coupled to the MS. The carrier gas was helium with a flow rate of 1.21 ml/min. The oven temperature was programmed as follows: 80°C for 1 min and subsequently held isothermal for 2 min, injector port: 260°C, detector: 280°C, split ratio 1 : 10, and volume injected: 1 *μ*l of the oil. The recording was performed at 70 eV, scan time 1.5 sec, and mass range 40–600 amu. Software adopted to handle mass spectra and chromatographs was a Chem station.

### 2.5. Identification of Chemical Constituents

The individual peaks/constituents were identified by GC-MS using comparison of their Kovats Index (K.I.) either with those of authentic compounds available in the author's laboratory or with those of literature in close agreement to K.I. Further identification was made by comparison of the fragmentation pattern of mass spectra obtained by GC-MS analysis with those stored in the spectrometer database of NBS 54 K.L, WILEY8 libraries. Retention indices of the components were determined a relative to the retention times of a series of n-alkanes a relative to C9–C20 on HPS and HP-20M columns.

### 2.6. Animals

Male Wistar rats, each with an approximate weight of 200–220 g, were kept in clean cages at 25 ± 2°C and in a diurnal cycle of 12 hours of light and 12 hours of darkness, with a relative humidity of 40–60 percent. The animals had access to water and food.

### 2.7. Preparation of Diabetic Animals

STZ (Pharmacia and Upjohn, USA) (60 mg/kg) solved in sterile saline just shortly prior to the test was intraperitoneally injected into rats. Animals with up to 180 milligrams/deciliter of the glucose level in their serum were used in the test five days after the injection [23].

### 2.8. Treatment Method

The wild thyme hydroalcoholic extract was intraperitoneally used as a treatment in different doses for 18 days. The number of animals in each group was 10. The first control group (Group (1) received merely regular food (normal diet: 10 kcal% fat, 20 kcal% protein, 70 kcal% carbohydrates, 3.75 kcal/g) and water. The second control group (Group 2) received saline daily, and the three experimental groups (Groups 3–5) daily and intraperitoneally received a low dose (100 mg), a medium dose (200 mg), and a high dose (400 mg) of wild thyme hydroalcoholic extract in two groups of healthy and diabetics for 18 days [24].

### 2.9. Assay of Hepatic Marker Enzymes

The hepatic marker enzymes such as AST, ALT, and ALP in the serum were measured using diagnostic kits (Parsazmon, Iran). As such, the glucose, cholesterol, and HDL cholesterol in serum, as well as LDL and very-low-density lipoprotein (VLDL) cholesterol were measured [25].

### 2.10. Histopathology

The liver tissue was fixed in 10% formalin for 48 h. It was then followed by dehydration by passing through a series of graded alcohol, beginning with 50% alcohol and progressing in graded steps to 100% (absolute) alcohol, and was finally embedded in paraffin. Liver slices (5-6 *μ*m thick) were prepared using a semiautomated rotator microtome, stained with Hematoxylin and Eosin dyes, and observed microscopically [23].

### 2.11. RT-qPCR Assays

Total RNA from liver tissues was extracted using the Column RNA Isolation Kit (DENAzist Asia Co., Iran) and reverse-transcribed with a cDNA synthesis kit (Thermo Fisher Scientific., USA).

The designed primers (Beacon Designer v8) were as follows: for Bcl-2, F: 5′-GAGCGTCAACAGGGAGA-3′ and R: 5′-GCCAGGAGAAATCAAACA-3′; for Bax, F: 5′-ACTAAAGTGCCCGAGCTGA-3′ and R: 5′-ACTCCAGCCACAAAGATGGT-3′; for C3, F: 5′-GGAGCTTGGAACGGTACGCT-3′ and R: 5′-AGTCCACTGACTTGCTCCCA-3′; for C9, F: 5′-AGCCAGATGCTGTCCCATAC-3′ and R: 5′-CAGGAGACAAAACCTGGGAA-3′; for *β*-actin: F: 5′-ATCAGCAAGCAGGAGTACGAT-3′ and R: 5′-AAAGGGTGTAAAACGCAGCTC-3′.

The normalization and analyses of the qPCR data were performed using Genex Version 6 software (MultiD, Göteborg, Sweden) and a Relative Expression Software Tool (REST; Qiagen, Hilden, Germany) [26].

### 2.12. Western Blotting

Total protein was extracted according to the instructions of the kit, and protein concentration was measured by the BCA protein quantification method (no. 23227; Pierce, Rockford, IL, USA). Protein samples were stored at −70°C before use. Electrophoresis was performed using Nu-PAGE 10% SDS-PAGE Bis-Tris gel in SDS-PAGE buffer. Polyvinylidene fluoride (PVDF) membrane was used for transfer. Next, the membrane was blocked with bovine serum albumin (3%). Afterwards, the membranes were washed with Tris-buffered saline containing tween 20 (TBST) and incubated overnight with primary antibodies (procaspase-3 (ab184787; Abcam Inc., Cambridge, MA, USA), procaspase-9 (ab184786; Abcam Inc., Cambridge, MA, USA), anti-Bcl-2 antibody (ab7973; Abcam Inc., Cambridge, MA, USA), anti-Bax (ab32503; Abcam Inc., Cambridge, MA, USA),and *β*-actin (sc-47778; Santa Cruz Biotechnology, Inc., Santa Cruz, CA, USA)) diluted 1 : 1000. After that, the membrane was washed three times with TBST and the secondary antibody (1 : 1000) was added to be incubated for 1 h and washed with TBST. Then, band intensities were detected using the chemiluminescent substrate Supersignal Femto kit and band densities were analyzed using the ImageJ 1.52a program (Bethesda, Maryland, USA) [27].

### 2.13. Statistical Data Analysis

The collected data were analyzed using SPSS 19 and GraphPad Prism 8 statistical software, and one-way analysis of variance (one-way ANOVA) and Tukey test at the significant level of *P* < 0.05 were run to investigate between-group differences. All results were presented as standard errors of mean (SEM).

## 3. Results

**Figure 1:**
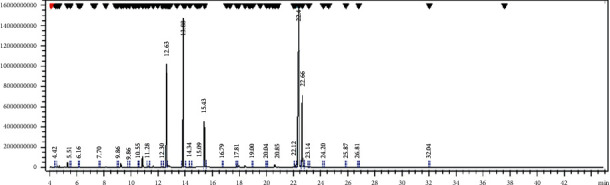
GC chromatogram of *Thymus vulgaris* (L) essential oil. Percentage data were obtained by gas chromatography-mass spectrometry (GC-MS).

The main compounds of thyme essential oil measured by GC/MS are shown in Table 1. Identification of compounds was done by comparing their mass spectra with their mass indices with the reference spectrum and also comparing their inhibition indices with the inhibition indices of these compounds. As shown, the four compounds of thymol (39.1%), p-cymene (20.63%), *γ*-terpinene (14.85%), and carvacrol (4.65%) constituted an aggregate of 86.54% of the composition of *T*. *vulgaris* essential oil (Figure 1).

### 3.1. Baseline Characteristics of the Study Cohort

**Figure 2:**
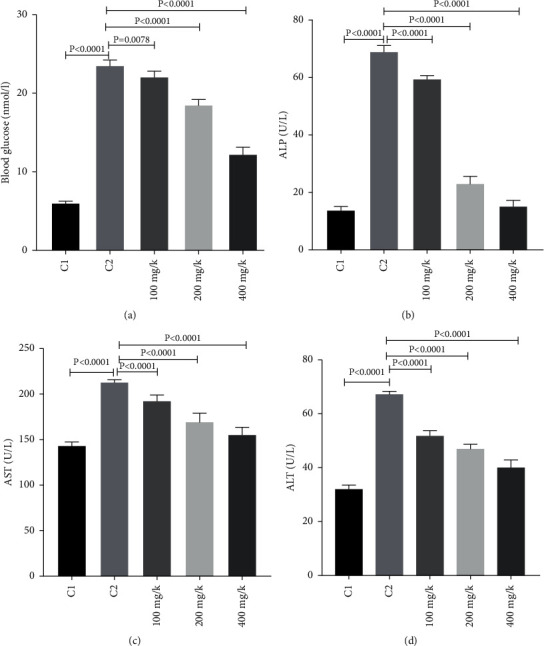
Effects of *Thymus vulgaris* on serum blood glucose (a), ALP (b), AST (c), and ALT (d) in control group (C1), diabetic group (C2), and 100 mg/k, 200 mg/k, and 400 mg/k of (STZ + *Thymus vulgaris*).

The results show that the level of serum glucose has significantly increased in diabetic rats compared to healthy rats (Figure 2(a)). However, serum glucose levels in the groups receiving thyme extract showed a significant decrease compared to the diabetic group (C2) (*P* < 0.0001). Regarding total serum cholesterol, a significant increase in the cholesterol level was observed in the diabetic control rats at the end of day 18 after the test compared to the control group 1 (Table 2). Thyme hydroalcoholic extract in the studied concentrations caused a significant reduction in total serum cholesterol levels compared to the diabetic control group (*P* < 0.05).

Serum levels of ALT, ALP, and AST in the diabetic group increased significantly compared with control group 1 (Figures 2(b)–2(d)). Also, serum levels of liver enzymes in diabetic groups treated with thyme extract significantly decreased compared to the diabetic group (*P* < 0.0001). Serum levels of liver enzymes in the diabetic group treated with thyme extract at a concentration of 400 mg/kg body weight significantly decreased, compared with the diabetic group treated with thyme extract at a concentration of 100 and 200 mg/kg body weight (*P* < 0.0001). Serum HDL-C levels decreased significantly in the diabetic group compared to the control group and serum LDL-C levels increased significantly (Table 2). However, in the groups treated with thyme, the levels of HDL-C and LDL-C increased and decreased significantly compared to the diabetic group, respectively (*P* < 0.05). Also, serum levels of VLDL in the diabetic group increased significantly compared to the control group 1 (*P* < 0.05). Moreover, serum VLDL levels in the diabetic group treated with thyme extract significantly decreased in comparison to the diabetic group (*P* < 0.05).

### 3.2. Pathology Results

**Figure 3:**
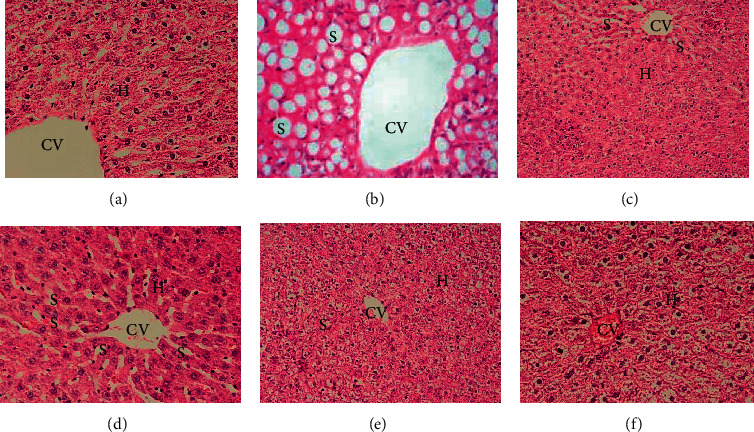
A photomicrograph of liver sections from (a) negative control rat showed normal structure of liver tissue and (b) control 2 (diabetic group) and (c)–(f) treated rats with extract and streptozotocin (STZ) showed normalization of liver tissue but with fine dilatation of main blood vessels and sinusoids. CVC: central venous catheter; H: hepatocyte; S: sinusoid (H&*E* ×200).

The results of the present study showed that the number of Kupffer inflammatory cells in the diabetic group increased significantly compared to the control group 11 (*P* < 0.001). Nonetheless, the number of blood vessels and hepatocytes in the diabetic group decreased significantly compared to the control group 1 (Figures 3(a) and 3(b)). A treatment of diabetic rats with thyme extract, in a dose-dependent manner, led to a significant increase in the number of blood vessels and hepatocytes and also a significant decrease in the number of Kupffer cells compared to the diabetic group (Figures 3(c)–3(f)). Comparison of the numbers of Kupffer cells, blood vessels, and hepatocytes between diabetic groups treated with thyme extract at concentrations of 100, 200, and 400 mg/kg body weight showed a statistically significant difference (*P* < 0.05). Histological examination indicated that the structure of the portal ducts and liver sinusoids was normal in control group 1, and no pathological changes were observed. In the diabetic group, inflammatory cells entered the lobule from the port space. Moreover, severe cell necrosis exists around the port space and there are scattered foci of necrosis in different parts of the liver lobules. Also, a noticeable decrease in the number of liver cells, changes in the structure, and irregularity of the liver sinusoids were observed. In the diabetic group treated with concentrations of 100 and 200 mg/kg of thyme extract, the rate of cell necrosis decreased, compared with the samples in the diabetic group. However, severe inflammation is still seen in the port area. In the diabetic group treated with a concentration of 400 mg/kg of thyme extract, a decrease in cell necrosis around the port space, a decrease in local inflammation of hepatocytes, and a decrease in hepatic sinusoids irregularity were observed, compared with diabetic samples in which hepatocytes have an almost normal structure.

### 3.3. Results of RT-qPCR Analysis

**Figure 4:**
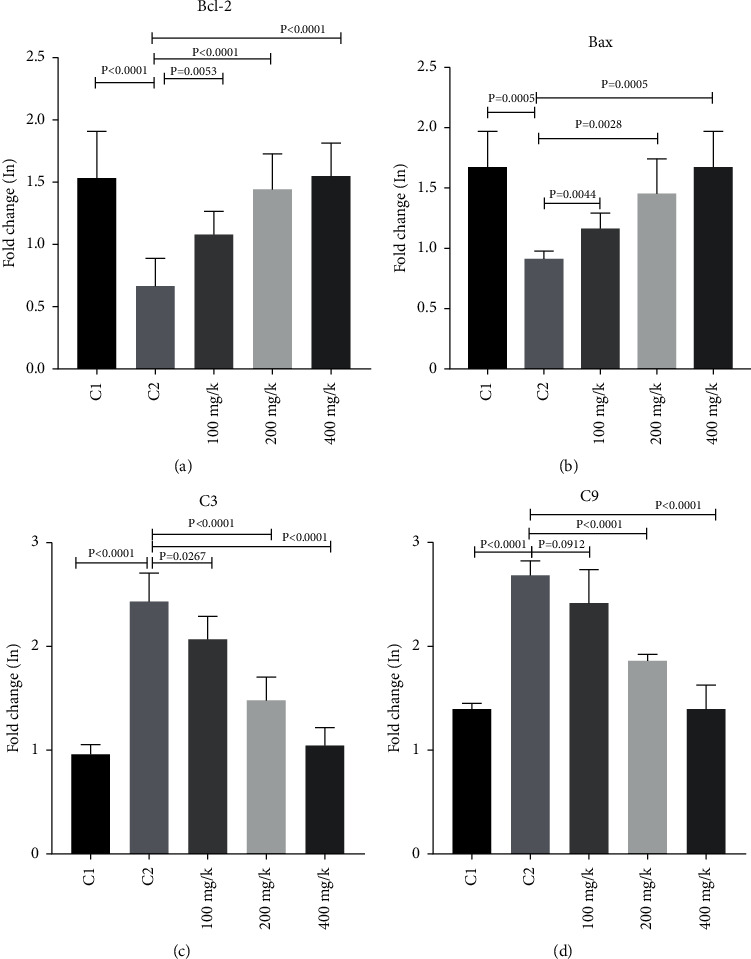
mRNA expression test results. RT-qPCR showed that the expression levels of four proteins in control group (C1), diabetic group (C2), and 100 mg/k, 200 mg/k, and 400 mg/k of streptozotocin (STZ)+*Thymus vulgaris*.

Bcl-2 gene expression significantly increased in the diabetic group compared to the control group 1, whereas Bax in the diabetic group significantly decreased compared to control group 1 (*P*=0.0005). On the other hand, in the groups receiving thyme extract, the expression of Bcl-2 decreased and Bax showed a significant increase, compared to the diabetic group without treatment (*P* < 0.0001). Nevertheless, the group receiving 100 mg/kg body weight of thyme did not show a statistically significant difference when compared to the control group 1. Furthermore, the expression of caspase 3 and 9 genes in the diabetic group without a treatment showed a significant increase compared to the control group (*P* < 0.0001). However, the expression level of caspase 3 and 9 genes in the diabetic groups treated with thyme extract showed a significant decrease compared to the diabetic control group (*P* < 0.0001) (Figure 4).

### 3.4. Results of Western Blot Analysis

**Figure 5:**
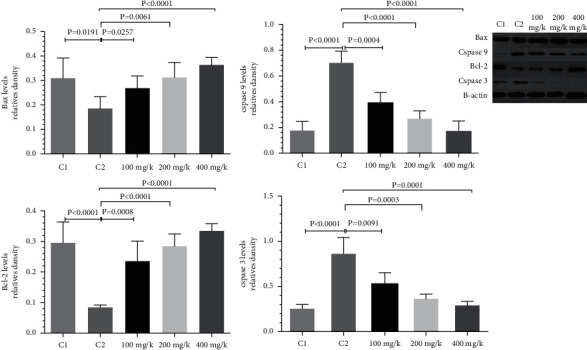
Protein expression test results. Western blot showed that the expression levels of four proteins in control group (C1), diabetic group (C2), and 100 mg/k, 200 mg/k, and 400 mg/k of streptozotocin ((STZ)+*Thymus vulgaris*).

Western blot results showed that the expression level of Bcl-2 in the diabetes + 400 mg/k *T*. *vulgaris* group (0.334 ± 0.104) was significantly higher than that in diabetes group (0.08 ± 0.018) (*P* < 0.0001); expression level of Bax protein in the diabetes + 400 *T*. *vulgaris* mg/k group (0.364 ± 0.1113) was significantly higher than that in diabetes group (0.186 ± 0.056) (*P* < 0.0001); caspase 3 protein in diabetes + 400 mg/k *T*. *vulgaris* group (0.294 ± 0.053) was significantly lower than that in diabetes group (0.863 ± 0.106) (*P* < 0.0001); and caspase 9 protein in the diabetes + 400 mg/k *T*. *vulgaris* group (0.174 ± 0.011) was significantly lower than that in diabetes group (0.701 ± 0.036) (*P* < 0.0001). Expression level of Bax protein in diabetes + SAH group (0.678 ± 0.124) was significantly higher than that in the blank control group (0.306 ± 0.086) and diabetes group (0.394 ± 0.098) (*P* < 0.05); and expression level of Bcl-2 protein in diabetes + SAH group (0.517 ± 0.062) was significantly higher than that in the blank control group (0.337 ± 0.116) and diabetes group (0.372 ± 0.185) (*P* < 0.05) (Figure 5).

## 4. Discussion

The present data suggest that *T*. *vulgaris* has a protective role in the liver injury caused by STZ-induced diabetes in rats, as demonstrated by blocking morphological and biochemical changes caused by this disease. The microscopic examination of liver tissue from STZ rats revealed loss of hepatic architecture, dilation of hepatic sinusoid capillaries close to the central vein, apoptotic hepatocytes and hepatocytes with lipid droplets in their cytoplasm indicating increased adipogenesis and cell death as well as signs of inflammation, which were all mitigated by *T*. *vulgaris*. In addition, STZ caused an increase in the size of the hepatocytes in their nuclei with no decrease in the nucleus-to-plasma ratio, as also found by others [28]. ALT, ALP, and AST enzymes are abundant in the liver, and any damage to liver cells increases their levels in the blood. These enzymes are used to evaluate hepatic disorders. An increase in the activity of the above enzymes reflects damage to the liver. Inflammatory disorders in hepatic cells lead to a sharp rise in transaminase levels [29, 30]. The results are in accordance with those of other studies in terms of the effects of wild thyme hydroalcoholic extract on hepatic cells and the reduction of AST, ALT, and ALP enzymes [31–33]. According to Yam (2007) and Janbaz (2004), it is proven that caffeic acid prevents the increase of serum enzymes and thus protects against methane tetrachloride-induced hepatic damage. These enzymes prevent liver protection activity through different mechanisms [34, 35]. ALT and AST enzymes decrease with the rise in the antioxidant activity of the liver [36]. According to Matsuura (2003) and Bozin (2006), it appears that due to their antioxidant properties, flavonoid compounds in wild thyme such as rosmarinic acid are able to neutralize free radicals of 1,1-diphenyl-2-picrylhyrazyl (DPPH) and prevent their destructive effects [37, 38].

**Figure 6:**
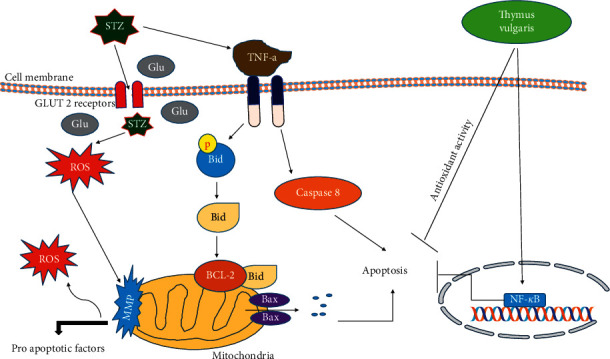
Schematic model depicting the mechanism of streptozotocin (STZ)-induced cytotoxicity in liver cells. STZ competes with glucose (Glu) to enter the cells via GLUT 2 receptors, causing Akt phosphorylation, which, in turn, causes further translocation of the GLUT 2 receptors. STZ also causes apoptosis by affecting the TNF-a receptor. The model also shows that STZ induces cytotoxicity and apoptosis through increased ROS/NOS production, oxidative/nitrosative stress, increased LPO, DNA damage, a decreased GSH/GSSG ratio, and mitochondrial dysfunction. Although *T*. *vulgaris* protects the liver cells from damage of STZ through NF-*κ*B pathways, *T*. *vulgaris* appears to be protective against STZ-induced apoptosis in liver cells through its potent antioxidant compounds.

*T*. *vulgaris* does not normalize hyperglycemia, suggesting the action of *T*. *vulgaris* on liver dysfunction does not pertain to systemic variables associated with glucose metabolism. *T*. *vulgaris*, nonetheless, tends to balance out dyslipidemia in rats with STZ-induced diabetes. *T*. *vulgaris* extract proved to have a hypolipidemic effect represented by the decrease in TC as well as LDLC and increase HDL-C levels. When compared to the control group 1, Bcl-2 gene expression had a significant increase in the diabetic group, while Bax decreased significantly in the diabetic group. Alternatively, when compared to the diabetic group that did not receive a treatment, the expression of Bcl-2 decreased and the expression of Bax increased significantly in the groups that received thyme extract. Moreover, as compared to the control group, the expression of caspase 3 and 9 genes were significantly higher in the diabetic group without a treatment. Nevertheless, as compared to the diabetic control group, the expression levels of caspase 3 and 9 genes in the diabetic groups that received thyme extract were significantly decreased. *T*. *vulgaris*' antiapoptotic potential is sufficient to restore the cells' normal conditions (Figure 6).

Furthermore, stimulation of DNA polymerase by flavonoid compounds causes an increase in rRNA synthesis and results in the reconstruction of hepatic cells [39, 40]. Based upon Oktem (2006), lithospermic B, 12-hydroxy jasmonic acid, ursolic acid, and other phenolic compounds reduce hepatic inflammation by inhibiting the lipo-oxygenize cycle and preventing leukotrienes and free radicals production in hepatic Kupffer cells in mice [41]. According to the results of Subten Ocak (2007), caffeic acid, one of the antioxidant compounds in thyme, prevents the high production of nitric oxide and reduces nephrotoxic-induced damage [42].

In summary, here we provide additional evidence and have confirmed that the mechanism of STZ-induced cytotoxicity and apoptosis in liver cells in the rat. All the abovementioned findings confirm the results obtained in this study. Thyme extract contains important antioxidant compounds such as thymol, which can prevent streptococcal damage in liver cells by reducing the expression of apoptosis-related factors. Analyses of liver enzymes reveal the notion that STZ can be beneficial for diabetic hepatitis by preventing liver damage and improving the morphology of liver tissue cells.

## Figures and Tables

**Table 1:** Main compounds of *Thymus vulgaris* essential oil.

Compound	Percent	RI	Chemical formula	RT	LRI	Identification
*α*-Pinene	1.7	930	C_10_H_16_	6.76	939	RI, MS
*α*-Thujene	1.41	928	C_10_H_16_	7.11	936	RI, MS
*β*-Pinene	0.64	979	C_10_H_16_	14.16	984	RI, MS
*α*-Terpinene	0.4	971	C_10_H_16_	11.61	985	RI, MS
Sabinene	0.51	964	C_10_H_16_	29.96	972	RI, MS
Borneol	0.32	1172	C_10_H_18_O	13.68	1180	RI, MS
Myrcene	1.64	871	C_10_H_16_	14.62	902	RI, MS
Thymol	39.1	2010	C_10_H_14_O	21.50	2015	RI, MS
Linalool	0.39	1080	C_10_H_18_O	12.53	1085	RI, MS
Carvacrol	4.65	1296	C_10_H_14_O	30.16	1330	RI, MS
*trans*-Sabinene hydrate	0.2	1050	C_10_H_18_O	15.36	1068	RI, MS
Pulegone	36.3	1214	C_10_H_16_O	18.11	1221	RI, MS
Bornyl acetate	0.9	1284	C_12_H_20_O_2_		1291	RI, MS
p-Cymene	20.63	1223	C_10_H_14_	35.40	1227	RI, MS
*γ*-Cadinene	0.1	1531	C_15_H_24_	38.60	1535	RI, MS
*γ*-Terpinene	14.85	1050	C_10_H_16_	11.16	1060	RI, MS

RI: retention indices calculated on apolar; RT: retention time (min); LRI: retention indices of literature.

**Table 2:** Lipid profile of streptozotocin-induced rat's liver injury treated with *T*. *vulgaris* leaves alcoholic extract.

Groups	TC	HDL-C	LDL-C	VLDL
Control 1	98.36 ± 2.96	62.5 ± 3.45	26.35 ± 2.1	20.72 ± 1.96
Control 2 (diabetic group)	120.45 ± 3.35	49.68 ± 3.39	31.12 ± 2.86	24.66 ± 2.14
100 mg/k (STZ + *Thymus vulgaris*)	115.25 ± 2.58	55.85 ± 2.98	29.36 ± 2.42	22.45 ± 1.86
200 mg/k (STZ + *Thymus vulgaris*)	109.18 ± 2.76	60.19 ± 3.56	27.56 ± 2.21	21.59 ± 1.79
400 mg/k (STZ + *Thymus vulgaris*)	102.12 ± 2.47	61.95 ± 2.15	26.78 ± 1.84	21.36 ± 1.44

Data are presented as the means ± SD of five replicates. Data were analyzed by *T*-test. *P* < 0.001: value with the same letter has no significant but value with a different letter has significant at 0.05. TC: total cholesterol; HDL-C: high-density lipoprotein cholesterol; VLDL-C: very low density lipoprotein cholesterol; LDL-C: low-density lipoprotein cholesterol.

## Data Availability

The datasets generated during and/or analyzed during the current study are available from the corresponding author on reasonable request.

## References

[1] Chawla R., Chawla A., Jaggi S. (2016). Microvasular and macrovascular complications in diabetes mellitus: distinct or continuum?. *Indian Journal of Endocrinology and Metabolism*.

[2] Prabodha L. B. L., Sirisena N. D., Dissanayake V. H. W. (2018). Susceptible and prognostic genetic factors associated with diabetic peripheral neuropathy: a comprehensive literature review. *International Journal of Endocrinology*.

[3] Samadi-Noshahr Z., Hadjzadeh M. A., Moradi-Marjaneh R., Khajavi-Rad A. (2020). The hepatoprotective effects of fennel seeds extract and trans-anethole in streptozotocin-induced liver injury in rats. *Food science & nutrition*.

[4] Bhatt H. B., Smith R. J. (2015). Fatty liver disease in diabetes mellitus. *Hepatobiliary Surgery and Nutrition*.

[5] Saraei P., Asadi I., Kakar M. A., Moradi-Kor N. (2019). The beneficial effects of metformin on cancer prevention and therapy: a comprehensive review of recent advances. *Cancer Management and Research*.

[6] Liu X., Liu W., Ding C. (2020). Antidiabetic effects of arginyl-fructosyl-glucose, a nonsaponin fraction from ginseng processing in streptozotocin-induced type 2 diabetic mice through regulating the PI3K/AKT/GSK-3*β* and bcl-2/bax signaling pathways. *Evidence-Based Complementary and Alternative Medicine*.

[7] Wu J., Yan L. J. (2015). Streptozotocin-induced type 1 diabetes in rodents as a model for studying mitochondrial mechanisms of diabetic *β* cell glucotoxicity. *Diabetes, Metabolic Syndrome and Obesity: Targets and Therapy*.

[8] Nita M., Grzybowski A. (2016). The role of the reactive oxygen species and oxidative stress in the pathomechanism of the age-related ocular diseases and other pathologies of the anterior and posterior eye segments in adults. *Oxidative Medicine and Cellular Longevity*.

[9] Raza H., John A. (2012). Streptozotocin-induced cytotoxicity, oxidative stress and mitochondrial dysfunction in human hepatoma HepG2 cells. *International Journal of Molecular Sciences*.

[10] Ito F., Sono Y., Ito T. (2019). Measurement and clinical significance of lipid peroxidation as a biomarker of oxidative stress: oxidative stress in diabetes, atherosclerosis, and chronic inflammation. *Antioxidants*.

[11] Chen L., Feng P., Peng A. (2020). Protective effects of isoquercitrin on streptozotocin-induced neurotoxicity. *Journal of Cellular and Molecular Medicine*.

[12] Rajappa R., Sireesh D., Salai M. B., Ramkumar K. M., Sarvajayakesavulu S., Madhunapantula S. V. (2019). Treatment with naringenin elevates the activity of transcription factor Nrf2 to protect pancreatic *β*-cells from streptozotocin-induced diabetes in vitro and in vivo. *Frontiers in Pharmacology*.

[13] Tan Y., Zhang Z., Zheng C., Wintergerst K. A., Keller B. B., Cai L. (2020). Mechanisms of diabetic cardiomyopathy and potential therapeutic strategies: preclinical and clinical evidence. *Nature Reviews Cardiology*.

[14] Butler A. E., Janson J., Bonner-Weir S., Ritzel R., Rizza R. A., Butler P. C. (2003). *β*-Cell deficit and increased *β*-cell apoptosis in humans with type 2 diabetes. *Diabetes*.

[15] Bae S., Siu P. M., Choudhury S. (2010). Delayed activation of caspase-independent apoptosis during heart failure in transgenic mice overexpressing caspase inhibitor CrmA. *American Journal of Physiology. Heart and Circulatory Physiology*.

[16] Mostafa Tork O., Ahmed Rashed L., Bakr Sadek N., Abdel-Tawab M. S. (2019). Targeting altered mitochondrial biogenesis in the brain of diabetic rats: potential effect of pioglitazone and exendin-4. *Reports of Biochemistry and Molecular Biology*.

[17] Björnsson E. S., Gu J., Kleiner D. E., Chalasani N., Hayashi P. H., Hoofnagle J. H. (2017). Azathioprine and 6-mercaptopurine induced liver injury: clinical features and outcomes. *Journal of Clinical Gastroenterology*.

[18] Abolghasemi R., Haghighi M., Solgi M., Mobinikhaledi A. (2019). Rapid synthesis of ZnO nanoparticles by waste thyme (thymus vulgaris L.). *International Journal of Environmental Science and Technology*.

[19] Ghorbani Ranjbary A., Ghorbani Ranjbary N., Asmarian S. H., Ghorbani Ranjbary Z. (2012). Effect of origanum vulgare hydroalcoholic extract on liver enzymes, cholesterol, triglycerides, cholesterol-hdl, cholesterol-ldl, total bilirubin, creatinine, albumin, total protein in rat. *Research Journal of Pharmaceutical, Biological and Chemical Sciences*.

[20] Uritu C. M., Mihai C. T., Stanciu G. D. (2018). Medicinal plants of the family lamiaceae in pain therapy: a review. *Pain Research and Management*.

[21] Ghorbani Ranjbary A., Ghorbani Ranjbary N., Ghorbani Ranjbary Z., Jouibar F. (2014). Effects of intraperitoneal injection of extracts of origanum vulgare on gonadotropin and testosterone hormones in male Wistar rats. *Journal of Babol University of Medical Sciences*.

[22] Ghorat F., Azizkhani M., Naji S., Ranjbary A. G., Doostishoar F. (2017). Histopathological evaluation of burdock (arctium lappa) root hydroalcoholic extract on wound healing. *Iranian Red Crescent Medical Journal*.

[23] Bahadori R., Moridi R., Ghorbani Ranjbary A. (2015). The study of milk containing lactobacillus acidophilus on histological and serum markers of liver tissue injury in streptozotoc-3. *Journal of Babol University of Medical Sciences*.

[24] Ghorbani Ranjbary A., Ranjbary N. G., Asmarian S. H., Ranjbary Z. G. (2014). Effect of origanum vulgare hydroalcoholic extract on liver enzymes, cholesterol, triglycerides, cholesterol-HDL, cholesterol-LDL, total bilirubin, creatinine, albumin, total protein in rat. *Journal of Pharmaceutical, Chemical and Biological Sciences*.

[25] Villanueva Bermejo D., Angelov I., Vicente G. (2015). Extraction of thymol from different varieties of thyme plants using green solvents. *Journal of the Science of Food and Agriculture*.

[26] Fecka I., Turek S. (2008). Determination of polyphenolic compounds in commercial herbal drugs and spices from lamiaceae: thyme, wild thyme and sweet marjoram by chromatographic techniques. *Food Chemistry*.

[27] Azimi M., Mehrzad J., Ahmadi A., Ahmadi E., Ghorbani Ranjbary A. (2021). Apoptosis induced by ziziphora tenuior essential oil in human colorectal cancer cells. *BioMed Research International*.

[28] Hasan K. M. M., Tamanna N., Haque M. A. (2018). Biochemical and histopathological profiling of wistar rat treated with brassica napus as a supplementary feed. *Food Science and Human Wellness*.

[29] Al Nahdi A. M., John A., Raza H. (2017). Elucidation of molecular mechanisms of streptozotocin-induced oxidative stress, apoptosis, and mitochondrial dysfunction in rin-5F pancreatic *β*-cells. *Oxidative Medicine and Cellular Longevity*.

[30] El-Newary S. A., Shaffie N. M., Omer E. A. (2017). The protection of thymus vulgaris leaves alcoholic extract against hepatotoxicity of alcohol in rats. *Asian Pacific Journal of Tropical Medicine*.

[31] Giannini E. G., Testa R., Savarino V. (2005). Liver enzyme alteration: a guide for clinicians. *Canadian Medical Association Journal*.

[32] Kooti W., Kafash-Farkhad N., Ghorbani Ranjbary A., Sharafi-Ahvazi N. (2016). The effect of celery (apium graveolens) on reproductive parameters in male wistar rat. *Avicenna journal of phytomedicine*.

[33] Al-Amoudi W. M. (2017). Protective effects of fennel oil extract against sodium valproate-induced hepatorenal damage in albino rats. *Saudi Journal of Biological Sciences*.

[34] Yam M. F., Basir R., Asmawi M. Z., Ismail Z. (2007). Antioxidant and hepatoprotective effects of orthosiphon stamineus benth. *The American Journal of Chinese Medicine*.

[35] Janbaz K. H., Saeed S. A., Gilani A. H. (2004). Studies on the protective effects of caffeic acid and quercetin on chemical-induced hepatotoxicity in rodents. *Phytomedicine*.

[36] Bampidis V. A., Christodoulou V., Florou-Paneri P. (2005). Effect of dietary dried oregano leaves on growth performance, carcase characteristics and serum cholesterol of female early maturing turkeys. *British Poultry Science*.

[37] Matsuura H., Chiji H., Asakawa C., Amano M., Yoshihara T., Mizutani J. (2003). DPPH radical scavengers from dried leaves of oregano (origanum vulgare). *Bioscience Biotechnology and Biochemistry*.

[38] Bozin B., Mimica-Dukic N., Simin N., Anackov G. (2006). Characterization of the volatile composition of essential oils of some lamiaceae spices and the antimicrobial and antioxidant activities of the entire oils. *Journal of Agricultural and Food Chemistry*.

[39] Gibson D. G., Glass J. I., Lartigue C. (2010). Creation of a bacterial cell controlled by a chemically synthesized genome. *Science*.

[40] Sauviac L., Niebel A., Boisson-Dernier A., Barker D. G., de Carvalho-Niebel F. (2005). Transcript enrichment of nod factor-elicited early nodulin genes in purified root hair fractions of the model legume medicago truncatula. *Journal of Experimental Botany*.

[41] Öktem F., Yilmaz H. R., Ozguner F. (2006). Methotrexate-induced renal oxidative stress in rats: the role of a novel antioxidant caffeic acid phenethyl ester. *Toxicology and Industrial Health*.

[42] Ocak S., Gorur S., Hakverdi S., Celik S., Erdogan S. (2007). Protective effects of caffeic acid phenethyl ester, vitamin C, vitamin E and N-acetylcysteine on vancomycin-induced nephrotoxicity in rats. *Basic and Clinical Pharmacology and Toxicology*.

[43] https://www.researchsquare.com/article/rs-569088/v1.

